# Proposed algorithm for management of patients with thyroid nodules/focal lesions, based on ultrasound (US) and fine-needle aspiration biopsy (FNAB); our own experience

**DOI:** 10.1186/1756-6614-6-6

**Published:** 2013-04-20

**Authors:** Zbigniew Adamczewski, Andrzej Lewiński

**Affiliations:** 1 Department of Endocrinology and Metabolic Diseases, Medical University of Lodz, Polish Mother’s Memorial Hospital – Research Institute, Rzgowska 281/289, Lodz, 93-338, Poland

## Abstract

**Background:**

The standard management in patients with thyroid nodules is to assess the risk of malignancy, based on cytological examination. On the other hand, there are thyroid patterns of ultrasound (US) image, associated with an increased risk of malignancy.

The aim of our study was to create a diagnostic algorithm that would employ both data from US examination (expressed by a total score, according to our scoring system) and FNAB results, classified according to Bethesda system (The Bethesda System for Reporting Thyroid Cytopathology - TBSRTC categories).

**Material and methods:**

100 thyroid cancer foci (94 papillary carcinomas, 4 medullary carcinomas, 2 undifferentiated carcinomas) and 100 benign focal lesions were selected during postoperative histopathological examination of thyroid glands excised during surgery from 111 patients. The corresponding US images of each lesion – performed in the course of preoperative diagnostics – were evaluated for the presence of seven (7) different features in US image, suggesting a malignant character of lesion, viz*.* vascularity, i.e., the increased central intranodular blood flows, microcalcifications, “taller-than-wide” orientation, solid composition, hypoechogenicity, irregular margin and either absence of peripheral halo or the presence of outer shell of uneven thickness, surrounding the lesion. The sensitivity, specificity, positive predictive values, negative predictive values and odds ratios for each US feature were calculated.

**Results:**

In US image of the analyzed cancer foci, we obtained the following values of odds ratio for each of the above mentioned features suggesting malignancy: “taller-than-wide” orientation - odds ratio - 301.0, microcalcifications - 24.67, increased intranodular vascularity - 20.44, hypoechogenicity - 18.61, irregular margins - 7.81, absence of halo - 5.88, and solid composition - 4.16.

Taking into account our own experience and the present data, in juxtaposition with the opinions of other authors, we propose a division of US features into 3 groups of different prognostic importance, expressed by a total score calculated based on our scoring system. Accordingly, microcalcifications, “taller-than-wide” orientation, the increased intranodular vascularity, and hypoechogenicity constitute one group - each of the features in this group is awarded 1 point. In turn, the characteristics of minor prognostic importance, such as irregular margin, absence of halo, solid composition, and large size (a diameter longer than 3.0 cm) - are associated with the granting 0.5 points each. The most important prognostic features – a rapid growth (enlargement) of nodules/focal lesions and a presence of pathologically altered lymph nodes are associated with the granting 3 points for each.

Our scoring system can be applied in order to better assessment of thyroid US patterns in whole. In patients with a total score ranging from 0 < 4 points there is US pattern of a low risk of malignancy, with ≥ 4 < 7 points - intermediate risk, and in patients with a score ≥ 7 points – a high risk in question.

**Conclusion:**

Complementary use of our scoring system and FNAB TBSRTC categories can help to make optimal clinical decisions as regards the selection of treatment strategy.

## Background

Common use of ultrasonography (US) in the diagnostics of thyroid diseases and of other diseases in the neck region caused rapid increase of the number of detected impalpable, asymptomatic lesions in the gland. According to some authors, it allows to visualize 10 times more US lesions than the number of palpable nodules detected during the physical examination [1-3]. It creates diagnostic and therapeutic dilemmas, dominated by the question: what is the most proper medical management – observation and careful monitoring of existing nodule, or – the opposite - referring the patients for surgery. While follow up, the most frequent way to proceed are the repeated US examination and a fine needle aspiration biopsy (FNAB). Even though the biopsy is considered by many doctors as a basis for further monitoring, performing FNAB of any identified lesions may not be prudent [4,5]. It happens that FNAB confirms the benign nature of the lesion and, nevertheless, it is quite frequently repeated (often many times) in spite of the fact that US image pattern does not change during a long-term observation. On the other hand, the surgical treatment in patients with obvious clinical signs and symptoms of malignant neoplastic disease is sometimes delayed because of unnecessary diagnostic procedures (e.g., due to awaiting for recommended time after which FNAB should be performed again). Thus, the endocrinologist - while diagnosing the thyroid nodule - cannot forget a question of fundamental importance – what is a bigger threat to the patients – observation, i.e., monitoring of lesion and live with the existing thyroid nodule or subjecting the patient to surgical treatment of his/her thyroid.

The aim of our study was to create an algorithm that would employ both data from US examination and FNAB results, classified according to Bethesda recommendations (The Bethesda System for Reporting Thyroid Cytopathology - TBSRTC categories), in order to optimise diagnostic and therapeutic management in case of nodules/US focal lesions in the thyroid [6].

## Methods

The algorithm was developed on the basis of prospective diagnostics in patients with nodules/US focal lesions of the thyroid, hospitalized in the Department of Endocrinology and Metabolic Diseases, in the time period from January 2008 to June 2012, who – then - were referred for surgery. At the same time, data of other authors on assessing the risk of thyroid malignancy, based on US characteristics, FNAB results, family medical history, and - above all - clinical signs and symptoms were considered.

100 thyroid cancer foci (94 papillary carcinomas, 4 medullary carcinomas, 2 undifferentiated carcinomas) and 100 benign focal lesions were selected during postoperative histopathological examination of thyroid glands excised during surgery carried out in 111 patients, aged 23 to 79 years (mean age – 57.0 years). The corresponding US images of each lesion – obtained in the course of preoperative diagnostics – were evaluated in order to assess seven (7) US characteristics, the most useful in differentiating benign and malignant lesions [7-10]. All examinations were performed by the same diagnostician, with extensive experience in thyroid US, using Toshiba Aplio XG US device with a linear probe PLT 1204 BT 12–18 MHz. An evaluation of focal lesions was carried out in the grey scale (B-mode), also with use of Power-Doppler method.

The following US features were subjected to analysis (for each characteristics - an appearance suggesting a malignant character of lesion is specified, followed by the respective image of the same feature, speaking for its benign nature):

1. Vascularity - defined as the presence of irregular chaotic intranodular hypervascularity; also images of a total absence of blood flow in hypoechogenic, solid lesions were included into that group; in contrast to the peripheral, subcapsular blood flows, suggesting benign lesions [11,12].

2. Calcifications - assessed as the presence of microcalcifications, also their coexistence with other forms of calcifications (e.g., dystrophic); in contrast to the absence of calcifications, the latter suggesting benign nature of lesions.

3. Orientation - “taller than wide” shape of lesion in transverse and/or longitudinal planes; in contrast to all other lesion shapes.

4. Composition - solid lesions, also mixed lesions with cystic component not exceeding 10% of the volume; in contrast to lesions with cystic parts greater than 10% of total volume and/or to solely cystic lesions.

5. Echogenicity - understood as hypoechogenicity, defined as a reduced (“darker”) echogenicity (similar to the echogenicity of muscles, surrounding the thyroid); in contrast to normal thyroid echogenicity (isoechogenicity) in benign lesions.

6. Margin - blurred and poorly defined; in contrast to well-differentiated regular margin.

7. Halo - the absence of halo, or the presence of outer shell (rim) of uneven thickness that surrounds the lesion; in contrast to the presence of thin, regular halo which speaks for a benign nature of lesion.

### Statistical analysis

The statistical analysis was performed with use of the Prism 5.0 software (GraphPad, La Jolla, USA). Fisher exact test was used to analyze distribution of the obtained results between the examined benign and malignant lesions. For each tested variable – the sensitivity, specificity, positive predictive values (PPV), negative predictive values (NPV) and odds ratios were estimated. Additionally, the 95% confidence intervals of these parameters were determined. The results were regarded as significant for p < 0.05.

## Results

The comparison of distribution of US characteristics, assessed in two (2) analyzed groups, showed the presence of significant statistical differences in the frequency of their occurrence, depending on the histopathological diagnosis (benign vs malignant lesions) (Table 1).

**Table 1:** Distribution of US characteristics in malignant and benign thyroid lesions; p values indicating a statistical significance are marked by bold

**Parameter**	**No. of cases (%)**	**Malignant lesions**	**Benign lesions**	**p value**
		**No. of cases (%)**	**No. of cases (%)**	
**Vascularity**				
Intranodular and increased or completely absent	150 (75.0)	96 (64.0)	54 (46.0)	**<0.0001**
Peripheral, subcapsular	50 (25.0)	4 (8.0)	46 (92.0)	
**Microcalcifications**				
Positive	128 (64.0)	93 (72.7)	35 (27.3)	**<0.0001**
Negative	72 (36.0)	7 (9.7)	65 (90.2)	
**Orientation**				
Taller-than-wide	88 (44.0)	86 (97.7)	2 (2.3)	**<0.0001**
Wider-than-tall or of other shape	112 (56.0)	14 (12.5)	98 (87.5)	
**Composition**				
Solid	173 (86.5)	94 (54.3)	79 (65.7)	**0.0032**
Mixed	27 (23.5)	6 (22.2)	21 (77.8)	
**Echogenicity**				
Hypoechogenic	106 (53.0)	84 (79.2)	22 (20.1)	**<0.0001**
Isoechogenic	94 (47.0)	16 (17.0)	78 (83.0)	
**Margins**				
Irregular	107 (53.5)	77 (71.9)	30 (28.1)	**<0.0001**
Well circumscribed	93 (46.5)	23 (24.7)	70 (75.3)	
**Peripheral Halo**				
Absent or irregular, thick	111 (55.0)	76 (68.0)	35 (32.0)	**<0.0001**
Present, thin and regular	89 (45.0)	24 (27.0)	65 (73.0)	

Table 2 presents the obtained values of sensitivity and specificity, also positive predictive values (PPV) and negative predictive values (NPV) of the analyzed parameters. Our results are comparable with data from reports of other authors [8,9,12-16].

**Table 2:** Values of sensitivity, specificity, PPV and NPV of the analyzed US characteristics (parameters)

**Parameter**	**Sensitivity (%)**	**Specificity (%)**	**PPV (%)**	**NPV (%)**
Vascularity	96 (90–99)	46 (36–56)	64 (56–72)	92 (80–98)
Microcalcifications	93 (86–97)	65 (54–74)	73 (64–80)	90 (80–96)
Orientation	86 (77–92)	98 (93–99)	97 (92–99)	87 (80–93)
Composition	94 (87–97)	21 (13–30)	54 (46–61)	77 (57–91)
Echogenicity	84 (75–90)	78 (68–85)	79 (70–86)	82 (73–89)
Margins	77 (67–84)	70 (60–78)	72 (62–80)	75 (65–83)
Peripheral Halo	76 (66–84)	65 (56–74)	68 (59–77)	73 (62–82)

The 95% confidence intervals are given in the brackets.

**Figure 1:**
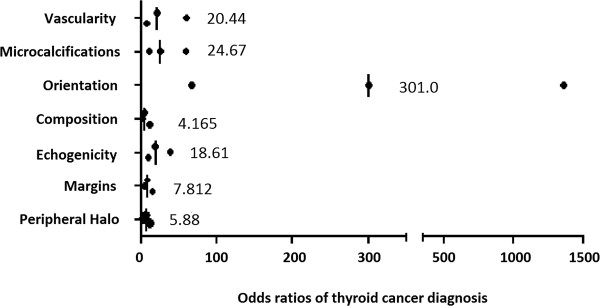
**Odds ratios of analyzed US characteristics in diagnostics of malignant thyroid lesions.** The values on the figure indicate the median odds ratio, with neighboring dots indicating the boundaries of 95% confidence intervals.

Simultaneously, the assessment of the odds ratios of thyroid cancer diagnosis, in the presence of the particular characteristics evaluated in each case, showed the increased risk of malignancy. The highest values of odds ratios were recorded in case of lesions "taller-than-wide" in shape in transverse and/or longitudinal planes, also the presence of microcalcifications, the incorrect pattern of vascularisation (intranodular and increased or completely absent) and/or hypoechogenicity were related to the increased cancer risk. This also applied - but to a lesser extent - to the lesions such as solid composition, the absence of halo, and blurred margins (Figure 1).

In addition, data from numerous reports of other authors indicate an increased risk of malignancy, especially in the case of:

the rapid growth of thyroid nodules/focal lesions [17] which is defined as a 20% increase in minimum two dimensions (at least - 2 mm) in the follow-up period shorter than 18 months [18];

thyroid lesions coexisting with lymphadenopathy that may suggest nodal metastases [19,20].

It is to be recalled that cervical US is the optimal method for searching lymph node metastases [18]. This examination allows to reveal more than 90% of the current metastases [21], the presence of which is diagnosed in approximately 25% microcarcinomas and even more frequently, up to 50% in larger tumours, with significant extrathyroidal extension, *viz.* T_4_ tumour stage in TNM scale [9,22]. However, it should be stressed that the simultaneous presence of the thyroid tumour and enlarged cervical lymph nodes does not necessarily constitute evidence of a direct causal relation;

the presence of thyroid tumours with a diameter exceeding 3–4 cm [23]; in tumours of larger size - follicular carcinomas are diagnosed more frequently [23,24]. In these cases - FNAB, used as the sole diagnostic test, does not allow formulation of the final diagnosis and confirmation of malignant nature of tumours and its result always must be evaluated with reference to clinical and US examinations.

Three characteristics listed above (rapid growth, enlarged lymph nodes, tumour size) have not been evaluated or subjected to statistical calculations in our present study for the reasons which we would like to explain. Follow-up of changes in the size of the analyzed lesions was not possible because the majority of US examinations were performed directly prior to surgery, and the patients submitted to surgical treatment were not previously hospitalised and diagnosed in our Department.

Due to the fact that the coexistence of thyroid lesions and of abnormal lymph nodes was found exclusively in case of malignant foci, we decided to exclude that feature from the statistical evaluation.

The diameter of all lesions examined by us did not exceed 3.0 cm, thus it was not possible to use the size of lesions for differentiation of the studied groups (malignant vs benign).

Taking into account our own observations, in juxtaposition with the opinions of other authors [4,5,19,20,25-27], we propose a division of US features into 3 groups of different prognostic importance. Accordingly, microcalcifications, “taller-than-wide” orientation, the incorrect pattern of vascularisation, and hypoechogenicity constitute one group - each of the features in this group is awarded 1 point. In turn, the characteristics of minor prognostic importance, such as irregular margin, absence of halo, solid composition, and large size (a diameter longer than 3.0 cm) - are associated with the granting 0.5 points each. The most important prognostic features – a rapid growth resulting in the enlargement of nodules/focal lesions, as well as a presence of pathologically altered lymph nodes are associated with the granting 3 points for each (Table 3).

**Table 3:** Summary of points awarded depending on the US characteristics

**Most important characteristic (each feature – 3 points)**	
G	Growth of nodules (resulting in rapid enlargement)
L	Pathologically altered lymph nodes
**Major prognostic characteristic (each feature – 1 point)**	
O	Orientation (shape)
C1	Calcifications (especially microcalcifications)
V	Vascularity (intranodular increased or absent)
E	Echogenicity (in particular hypoechogenicity)
**Minor prognostic characteristic (each feature – 0.5 point)**	
M	Margin (blurred and poorly defined)
H	Halo (absent or irregular and thick)
C2	Composition (solid)
D	Diameter (longer than 3.0 cm)

Having assigned the points for each characteristics, we created the scoring system that is applied for better assessment of thyroid US patterns in whole.

A total score constitutes the basis to assign the patients to groups of varying degree of the risk of malignancy. Accordingly, patients with a total score ranging from 0 to less than 4 points belong to the group of low risk US pattern, patients with a score from 4 to less than 7 points represent US pattern of intermediate risk of malignancy, and in patients with a score 7 points and more – US pattern of a high risk of malignancy occurs (Table 4).

**Table 4:** The scoring system of US patterns, applied for thyroid nodules/focal lesions

**Risk of malignancy, as assessed on the basis of US pattern**	**A total score**
Low risk	0 < 4 points
Intermediate risk	≥ 4 < 7 points
High risk	≥ 7 points

**Figure 2:**
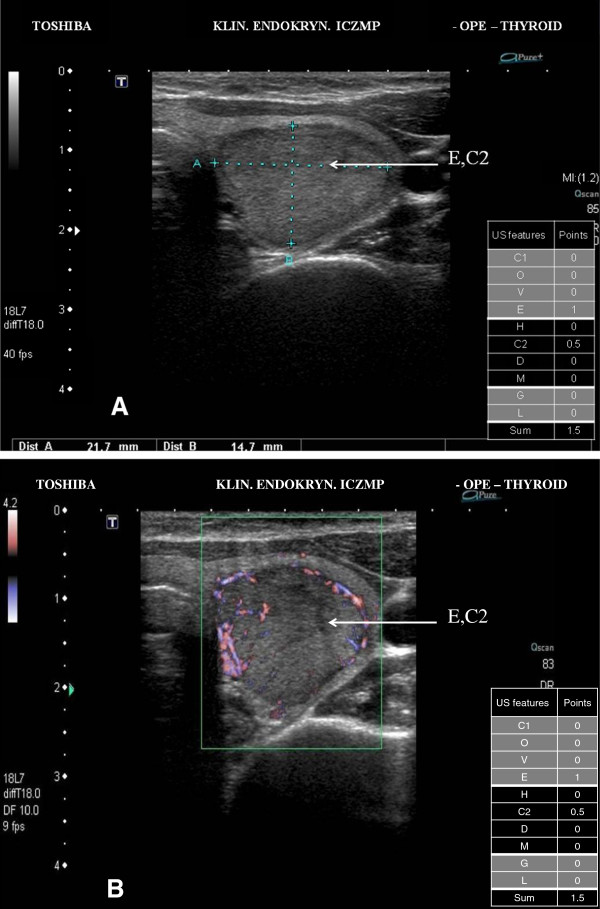
**Low risk thyroid nodule/focal lesion US pattern. A.** Ultrasound picture of the thyroid lesion (B-mode). The scoring system – 1.5 points (low risk US pattern 0 < 4 points); E – hypoechogenicity; C2 – solid composition; FNAB cytology in this case - category II TBSRTC; management - observation and repeat FNAB examination after 18 months. **B.** Ultrasound picture of the thyroid lesion (Power Doppler), peripheral blood flows.

**Figure 3:**
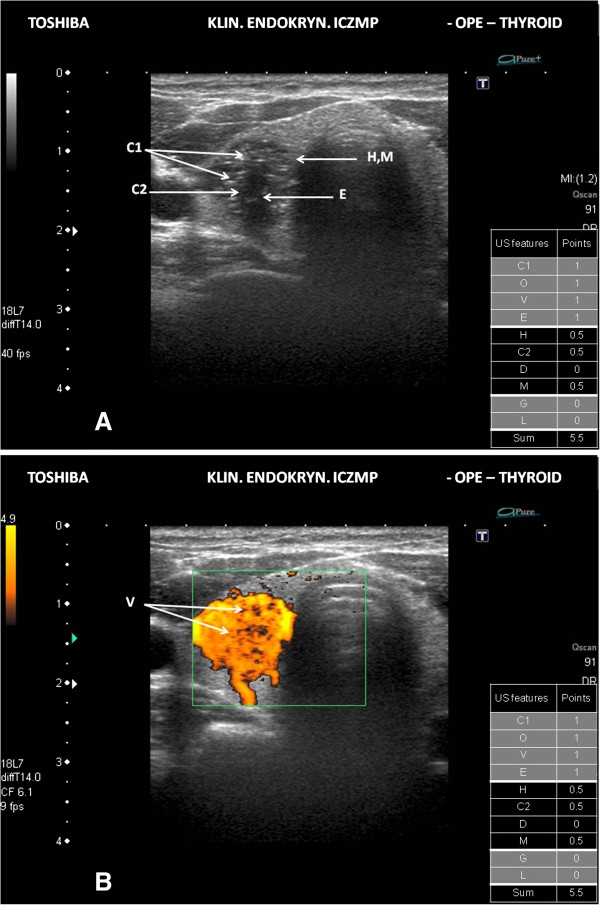
**Intermediate risk thyroid nodule/focal lesion US pattern. A.** Ultrasound picture of the thyroid lesion (B-mode). The scoring system - 5.5 points (intermediate risk US pattern ≥ 4 < 7 points); C1 – microcalcifications; O – orientation - “taller-than-wide” shape – not shown in the figure; E – hypoechogenicity; H – the absence of “halo”; C2 – solid composition; D – diameter - below 3 cm (not shown); M – irregular margin; FNAB cytology in this case - category IV TBSRTC; management - recommend surgery. **B.** Ultrasound picture of the thyroid lesion (Power Doppler). The scoring system - 5.5 points (intermediate risk US pattern ≥ 4 < 7 points); V – the increased intranodular blood flows.

**Figure 4:**
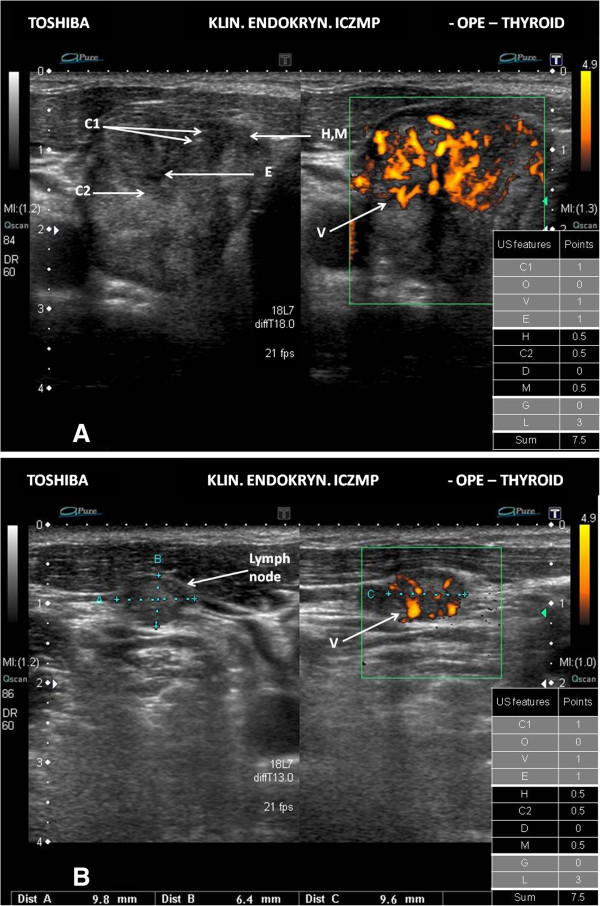
**High risk thyroid nodule/focal lesion US pattern. A.** Ultrasound picture of the thyroid lesion (B-mode, Power Doppler). The scoring system - 7.5 points (high risk US pattern ≥ 7 points); C1 – microcalcifications; O – orientation – shape other than “taller-than-wide” – not shown in the figure; V - the increased intranodular blood flows; E – hypoechogenicity; H – the absence of “halo”; C2 – solid composition; D – diameter - below 3 cm (not shown); M – irregular margin; FNAB cytology in this case - category V TBSRTC; management - recommend surgery. **B.** Ultrasound picture of the metastatic lymph node in the same case (B-mode, Power Doppler). The scoring system - 7.5 points (high risk US pattern ≥ 7 points); the increased blood flows, mainly peripheral, in the metastatic lymph node.

We have illustrated the practical application of our scoring system of the thyroid US patterns in Figures 2A, 2B, 3A, 3B, 4A and 4B.

## Discussion

It is well known that none of the individual US features allows to differentiate malignant from benign thyroid lesions. However, finding in US image of nodule/focal lesion one (1) or more than one suspicious features, correlates well with the risk of malignancy. This fact is commonly used for the qualification of patients with nodules/focal lesions for FNAB cytology.

The current system of cytological diagnoses (TBSRTC) greatly facilitates therapeutic decisions (Table 5) [6]. This applies mainly to diagnoses of malignant lesions (category VI TBSRTC) or a suspicion of malignancy (category V TBSRTC) which both create the need for urgent referral for surgery, in order to perform a total thyroidectomy, except cases inoperable at the time of diagnosis.

**Table 5:** **The Bethesda System for Reporting Thyroid Cytopathology (TBSRTC) – according to Cibas and Ali**[6]

**Diagnostic category**	**Risk of malignancy (%)**
I Nondiagnostic or unsatisfactory	1-4
II Benign	0-3
III Atypia of undetermined significance or follicular lesion of undetermined significance	5-15
IV Follicular neoplasm or suspicious for a follicular neoplasm	15-30
V Suspicious for malignancy	60-75
VI Malignant	97-99

Significantly more difficulties with making a therapeutic decision appear when FNAB belongs to the category IV TBSRTC - "follicular neoplasm or suspicious for a follicular neoplasm " or to category III TBSRTC - "follicular lesion of undetermined significance/atypia of undetermined significance". These diagnoses require a completely different clinical approach. Category IV TBSRTC is believed to be a final result, confirmed by the opinion of two independent pathologists. Repeating FNAB in such cases is not expected to provide additional benefits, thus, a repeated FNAB should be performed only in case of changes in the US pattern of lesions. The diagnosis of category III TBSRTC is "a category of exclusion" and should be treated as an optional diagnosis, thus, it can only ultimately be used. The main difference - when compared to category IV - is that category III requires a repeat biopsy, like in case nondiagnostic or unsatisfactory FNAB results (category I TBSRTC). One cannot forget that even in the case of benign lesions (category II TBSRTC) false-negative results are still possible and a risk of malignancy in such cases reaches up to 3% [6].

However, it should be emphasized that the thyroid US and FNAB cytology are diagnostic tests that should not always decide about the fate of our patient and about recommending him/her the surgical treatment. Many clinical parameters have long been recognized as predictors of malignancy in patients with thyroid nodules/lesions. Such factors include family medical history (genetic background), age younger than 20 and older than 60 years, irradiation of the neck and head area (particularly in the childhood) and male sex [4,18,28]. Moreover, certain clinical circumstances, e.g., if the patient complains of hoarseness (which is not a result of *stricte* laryngeal disease), or of ache in the tumour and dysphagia - usually caused by a firm thyroid tumour, may indicate a malignant character of lesion and may require an inquisitive intense diagnostics of any type, and appropriate treatment, in most cases of the radical type.

**Figure 5:**
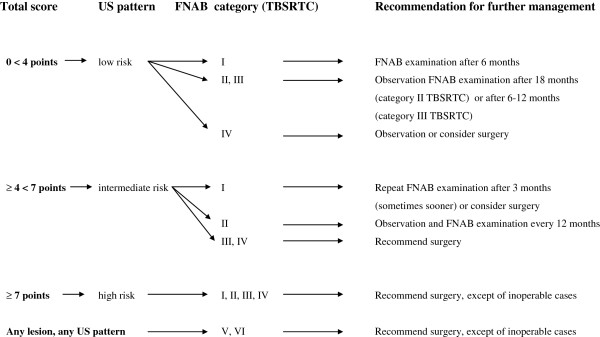
The algorithm of diagnostic and therapeutic management in thyroid nodules/focal lesions.

Based on the results of our observations, in combination with data of other authors, we decided to stratificate the risk of malignancy, depending on the ascertained signs and symptoms, US features, FNAB results according to TBSRTC categories, and – in consequence - we created the algorithm of diagnostic and therapeutic management, the use of which seems to be most favourable for patients with thyroid problems (nodules/focal lesions) (Figure 5).

We are aware of the fact that the assumption underlying of this study, namely comparison of distribution of US characteristics in the two groups of the same number of examined lesions, does not apply in the real epidemiological situation. However, we believe that such a design of the study highlights the most important US characteristics, the presence of which correlates - to the greatest extent - with the risk of malignancy. The presented algorithm of management in patients with thyroid problems is designed to highlight the complementarity of diagnostic procedures, such as US and FNAB. Simultaneously, we would like to stress that US is not only a tool used for selection of nodules which should be examined by FNAB but is also a diagnostic method, the outcomes of which can constitute a basis for the final therapeutic decision.

## Conclusions

The presented algorithm, as many other previously developed to assess the nature of thyroid lesions, does not solve all the possible encountered clinical problems. However, by taking into consideration the important US characteristics, related to an increased risk of malignancy, in combination with cytological assessment of lesions, at the same time - by paying attention on the significance of clinical data from medical history and physical examination, it fulfils expectations in daily clinical practice and is helpful in making the proper diagnostic and therapeutic decisions.

## Competing interests

The authors declare that they have no competing interests.

## Authors’ contributions

ZA designed the study, performed US examinations and participated in writing a manuscript. AL was involved in coordination of the study and supervised preparation of the final version of manuscript. Both authors read and approved the final manuscript.
